# Re-thinking preclinical models of cancer metastasis

**DOI:** 10.18632/oncoscience.450

**Published:** 2018-08-22

**Authors:** Maria F. Ullo, Jeremy S. Logue

**Affiliations:** Department of Regenerative and Cancer Cell Biology, Albany Medical College, Bldg. Medical Sciences (MS), Albany, NY 12208, USA

**Keywords:** metastasis, cell migration, mesenchymal, amoeboid, bleb, cytoskeleton, Src

Cell migration is a key step within the metastatic cascade and is the leading cause of death in cancer patients. Classically, focal adhesions are required at the leading edge of a motile cell. Wherein, focal adhesion formation requires the coordinated action of integrins, scaffolding, cytoskeletal regulators, and kinases. Src, the prototypical oncogene is known to facilitate adhesion formation by forming a signaling module with FAK. Because of this, Src has been the target of recent small molecule inhibitors. Our recent work however, shows that Src is differentially required in cells undergoing adhesion-independent migration. Thus, providing a possible explanation for the lack of efficacy observed in Src inhibitor clinical trials.

The activation of cellular Src kinase or c-Src is frequently observed in malignant cells. Its substrates include actin cytoskeleton regulators such as Eps8, C3G, and FAK. Upon adhesion maturation, the SH2 domain of Src recognizes an autophosphorylated site on FAK, leading Src to phosphorylate two additional sites and fully activate FAK. FAK can then go on to phosphorylate other focal adhesion proteins. In support of this role, numerous studies of adhesion-based cell migration have shown Src to be required. Accordingly, the pharmaceutical industry developed inhibitors of Src that were expected to reduce metastatic tumor burden. This unfortunately was not the case, as progression free survival in the metastatic setting for several cancers including melanoma were unaffected by the Src inhibitors Dasatinib or Saracatinib. These results led to the discontinuation of Saracatinib, whereas Dasatinib was approved for the treatment of CML owing to its ability to target the BCR-Abl fusion protein.

The aforementioned results are in fact reminiscent of the earlier MMP inhibitor trials. By degrading the ECM in front of an advancing cell, MMPs were thought to be essential and so these inhibitors were similarly expected to reduce metastasis. Clinical trials showed these inhibitors to be ineffective and for some cancers, they actually decreased survival [1]. Subsequently, using high-resolution imaging it was shown that cells given MMP inhibitors could undergo a Mesenchymal to Amoeboid Transition (MAT) [2]. Remarkably, some cells given MMP inhibitors were more aggressive as measured by invasion assays. The precise mechanism by which MMP inhibitors induce MAT is unknown, but high actomyosin contractility was shown to be required for amoeboid migration. In this work, amoeboid migration was characterized by cells having blebs, which require high contractility. Subsequently, intravital imaging confirmed that cancer cells could use blebs to migrate in tissues [3]. Thus, motile cancer cells can circumvent interventions by switching their migration mode.

More recently, it was shown that the choice between mesenchymal or bleb-based migration can be influenced by the *in vivo* cellular environment. More specifically, transformed cells exposed to microfabricated devices that mimic the confines of tissues can undergo a switch to “fast amoeboid,” “stable bleb,” or “Leader Bleb- Based Migration (LBBM)” [4-6], as this phenomenon was independently discovered by three different research groups. As we termed it LBBM [6], we refer to this phenomenon here by that name. In contrast to the circumferential blebs described in earlier studies, LBBM is characterized by the formation of a single very large and stable bleb that leads the cell forward. An important feature of LBBM is that it only requires non-specific friction with the extracellular environment [7]. At the heart of a leader bleb, is a cortical actin flow driven by myosin. It is not known how a leader bleb is spontaneously formed, but what is clear is that its formation is stimulated by cell confinement [4]. Additionally, intravital imaging has confirmed that cells can use LBBM to migrate in Zebrafish embryos [5].

In our recent work, we set out to determine the oncogenic signaling pathways that regulate the transition to LBBM [8]. To do this, we performed a targeted screen with clinically useful inhibitors. We found that treating melanoma cells with the Src inhibitor Dasatinib induced de-adhesion and blebbing. This result led us to speculate that LBBM and adhesion-based migration might require different signaling pathways. Remarkably, we found that LBBM is refractory to Dasatinib and a dominant negative SrcK295R. In contrast to the focal adhesions in adherent cells, a reporter of phosphotyrosine signaling showed a diffuse pattern in cells undergoing LBBM. Contrary to what was expected, an Atomic Force Microscopy (AFM) assay demonstrated that Dasatinib reduced cortical tension and intracellular pressure. At the same time, Dasatinib reduced cortical actin density and so we speculated that reductions in mechanical properties were circumvented by changes in the cortical actin network. Additionally, we evaluated the impact of high Src activity on LBBM by expressing a constitutively active Src^Y529F^ mutant. In adherent cells, Src^Y529F^ promoted focal adhesion and protrusion formation, whereas Src^Y529F^ expression led to chaotic blebbing in confined cells. Moreover, these cells moved slower and in a rather non-directional fashion when compared to LBBM. The Rac GEF C3G is a substrate of Src, in line with this we found that Rac and arp2/3 were activated in blebbing cells. Treating cells with an arp2/3 inhibitor reverted Src^Y529F^ expressing cells back to LBBM. Thus, our study revealed that Src is differentially required by LBBM and adhesion-based migration.

Relative to focal adhesion-based migration, bleb- based or adhesion-independent migration is understudied. Given the results of clinical trials and studies described here, progress will require careful consideration of the *in vivo* cellular environment and bleb-based migration. The continued improvement of assays that mimic the confines of tissues combined with high-resolution imaging will be invaluable to this effort.

## References

[1] Coussens LM (2002). Science.

[2] Wolf K (2003). J Cell Biol.

[3] Tozluoglu M (2013). Nat Cell Biol.

[4] Liu YJ (2015). Cell.

[5] Ruprecht V (2015). Cell.

[6] Logue JS (2015). Elife.

[7] Bergert M (2015). Nat Cell Biol.

[8] Logue JS (2018). Oncogene.

